# 8a-Methyl-5,6,8,8a,9,10-hexa­hydro-10,12a-epoxy­isoindolo[1,2-*a*]isoquinolinium iodide

**DOI:** 10.1107/S1600536810017903

**Published:** 2010-05-19

**Authors:** Flavien A. A. Toze, Julya D. Ershova, Mykola D. Obushak, Fedor I. Zubkov, Victor N. Khrustalev

**Affiliations:** a Department of Chemistry, University of Douala, Faculty of Sciences, PO Box 24157, Douala, Republic of Cameroon; b Department of Organic Chemistry, Russian People’s Friendship University, 6 Miklukho-Maklaya St, Moscow 117198, Russian Federation; c Department of Organic Chemistry, Ivan Franko National University of Lviv, 6 Kyryla and Mefodiya St, Lviv 79005, Ukraine; d X-Ray Structural Centre, A. N. Nesmeyanov Institute of Organoelement Compounds, Russian Academy of Sciences, 28 Vavilov St, B-334, Moscow 119991, Russian Federation

## Abstract

The title compound, C_17_H_18_NO^+^·I^−^, is an adduct resulting from an intra­molecular Diels–Alder reaction of methallyl chloride with 3,4-dihydro-1-furylisoquinoline. The cation comprises a fused penta­cyclic system containing three five-membered rings (dihydro­pyrrole, dihydro­furan and tetra­hydro­furan) and two six-membered rings (tetra­hydro­pyridine and benzene). The five-membered rings have the usual envelope conformations, and the central six-membered tetra­hydro­pyridine ring adopts the unsymmetrical half-boat conformation. In the crystal, cations and iodide anions are bound by weak inter­molecular hydrogen-bonding inter­actions into a three-dimensional framework.

## Related literature

For general background to the method proposed by our group for obtaining hydrogenated isoindolo[2,1-*a*]isoquinolines using commercially available furfurals and phenethyl­amines as starting materials, see: Zubkov *et al.* (2004 ▶); Boltukhina *et al.* (2006 ▶). For related structures, see: Tagmazyan *et al.* (1976 ▶, 1977 ▶); Ahmad *et al.* (1987 ▶); Rasheed *et al.* (1991 ▶); Zubkov *et al.* (2009 ▶).
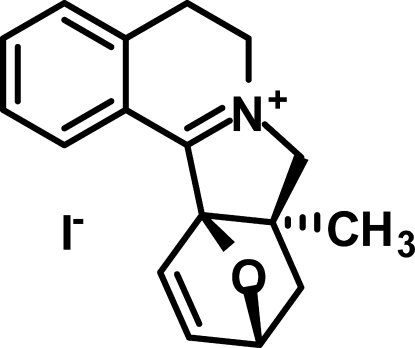


## Experimental

### 

#### Crystal data

C_17_H_18_NO^+^·I^−^
*M* *_r_* = 379.22
Monoclinic, 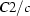

*a* = 15.5047 (6) Å
*b* = 8.0757 (3) Å
*c* = 25.1874 (12) Å
β = 104.204 (1)°
*V* = 3057.3 (2) Å^3^
*Z* = 8
Mo *K*α radiation
μ = 2.09 mm^−1^
*T* = 100 K
0.30 × 0.20 × 0.15 mm

#### Data collection

Bruker APEXII CCD diffractometer
Absorption correction: multi-scan (*SADABS*; Sheldrick, 2003 ▶) *T*_min_ = 0.573, *T*_max_ = 0.745
18881 measured reflections
4450 independent reflections
4123 reflections with *I* > 2σ(*I*)
*R*_int_ = 0.031

#### Refinement

*R*[*F* ^2^ > 2σ(*F* ^2^)] = 0.025
*wR*(*F* ^2^) = 0.054
*S* = 1.00
4450 reflections
182 parameters
H-atom parameters constrained
Δρ_max_ = 0.48 e Å^−3^
Δρ_min_ = −0.94 e Å^−3^

### 

Data collection: *APEX2* (Bruker, 2005 ▶); cell refinement: *SAINT-Plus* (Bruker, 2001 ▶); data reduction: *SAINT-Plus*; program(s) used to solve structure: *SHELXTL* (Sheldrick, 2008 ▶); program(s) used to refine structure: *SHELXTL*; molecular graphics: *SHELXTL*; software used to prepare material for publication: *SHELXTL*.

## Supplementary Material

Crystal structure: contains datablocks global, I. DOI: 10.1107/S1600536810017903/rk2205sup1.cif

Structure factors: contains datablocks I. DOI: 10.1107/S1600536810017903/rk2205Isup2.hkl

Additional supplementary materials:  crystallographic information; 3D view; checkCIF report

## Figures and Tables

**Table 1:** Hydrogen-bond geometry (Å, °)

*D*—H⋯*A*	*D*—H	H⋯*A*	*D*⋯*A*	*D*—H⋯*A*
C2—H2⋯I1^i^	0.95	3.23	3.999 (2)	139
C6—H6*A*⋯I1^ii^	0.99	3.10	4.028 (2)	157
C8—H8*A*⋯I1^iii^	0.99	3.04	3.942 (2)	153
C8—H8*B*⋯I1^ii^	0.99	3.17	4.072 (2)	153
C9—H9*B*⋯I1	0.99	3.10	3.862 (2)	135
C12—H12⋯I1^iv^	0.95	3.20	3.862 (2)	128
C13—H13*B*⋯I1^iv^	0.98	3.21	3.970 (2)	136

Symmetry codes: (i) 
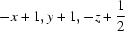
; (ii) 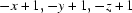; (iii) 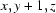; (iv) 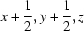.

**Fig. 1.**
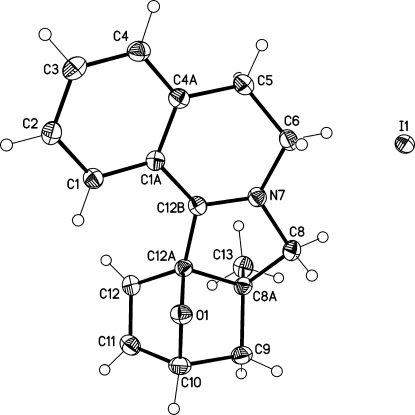
Molecular structure of I. Displacement ellipsoids are shown at the 50% probability level. H atoms are presented as a small spheres of arbitrary radius.

**Fig. 2.**
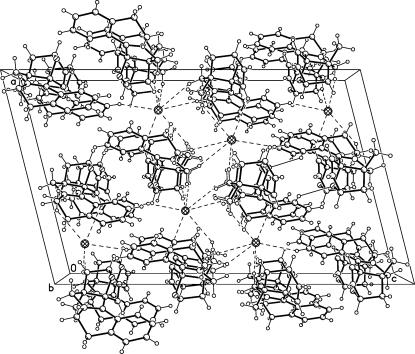
Crystal packing of I along the b axis. Dashed lines indicate the weak intermolecular C—H···I hydrogen bonding interactions.

**Fig. 3.**
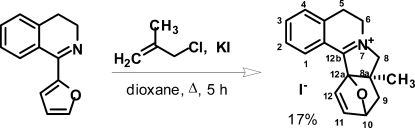
Tandem alkylation/[4+2] cycloaddition reaction of methallyl chloride with 3,4-dihydro-1-furylisoquinoline.

## supplementary crystallographic information

### Comment

Recently our group has proposed an efficient approach (Zubkov *et al.*, 2009) to potentially bioactive substances - hydrogenated isoindolo[2,1-*a*]isoquinolines using commercially available furfurals and phenethylamines as starting materials (Boltukhina *et al.*, 2006). The intramolecular furan Diels–Alder reaction (*IMDAF*) (Zubkov *et al.*, 2004) between unsaturated acid derivatives and the furan core of the amines was the key step of the transformations mentioned above.

Trying to apply our chemistry to target natural products, we were attracted to isoindoloisoquinoline alkaloids. Today, there are three known natural alkaloids containing the isoindolo[2,1-*a*]isoquinoline skeleton - *Nuevamine, Jamtine* (and its *N*-oxide) and *Hirsutine* (Rasheed *et al.*, 1991).

This work realizes aforementioned approach and describes the structure of an alkaloid-like *IMDAF* product I containing the nodal iminium nitrogen atom (Tagmazyan *et al.*, 1976; Ahmad *et al.*, 1987).

Compound I, [C_17_H_18_NO^+^][I^-^], is the adduct of intramolecular Diels–Alder reaction of methallyl chloride with 3,4-dihydro-1-furylisoquinoline. The cation of I comprises a fused pentacyclic system containing three five-membered rings (dihydropyrrole, dihydrofuran and tetrahydrofuran) and two six-membered rings (tetrahydropyridine and benzene) (Fig. 1). The five-membered rings have usual *envelope* conformations, and the central six-membered tetrahydropyridine ring adopts the unsymmetrical *half*-*boat* conformation. The nitrogen N7 atom has a trigonal-planar geometry (sum of the bond angles is 359.5°). The dihedral angle between the planes of the dihydropyrrole (N7/C8/C12A/C12B) and benzene rings is 20.1 (1)°.

The cation of I possesses three asymmetric centers at the C8A, C10 and C12A carbon atoms and can have potentially eight diastereomers. The crystal of I is racemic and consists of enantiomeric pairs with the following relative configuration of the centers: *rac*-8 A*R**,10*R**,12 A*R**.

The crystal packing of the cations of I is stacking along the *b* axis (Fig. 2). In the crystal, the cations and iodide anions are bound by the weak intermolecular hydrogen bonding interactions into 3-dimensional framework (Fig. 2, Table 1).

### Experimental

Potassium iodide (3.1 g, 11 mmol) and methallylchloride (0.8 ml, 8.25 mmol) were added to a solution of 1-furyl-3,4-dihydroisoquinoline (1.08 g, 5.5 mmol) in dioxane (50 ml). The reaction mixture was refluxed for 5 h (monitoring by *TLC* until disappearance of the starting compound sport). At the end of the reaction, solvent was removed under reduced pressure and the residue was crystallized from ethyl acetate–ethanol mixture to give 0.35 g of isoquinolinium iodide as brown prisms (Fig. 3). Yield is 17%. The single crystals of product I were obtained by slow crystallization from acetonitrile (yield 73%). M.p. = 451–453 K. R*_f_* = 0.3 (ethyl acetate–ethanol, 4:1). IR (KBr), ν/cm^-1^: 1632, 2361, 2947, 3399. ^1^H NMR (CDCl_3_, 400 MHz, 300 K): δ = 1.33 (s, 3H, *Me*), 1.38 (d. 1H, H9 (*endo*), J_9A,9B_ = 11.8), 2.50 (dd, 1H, H9 (*exo*), J_9(_*_exo_*_),10_ = 4.4, J_9A,9B_ = 11.8), 3.33 (ddd, 1H, H5B, J_5B,6A_ = 2.5, J_5B,6B_ = 5.6, J_5A,5B_ = 16.8), 4.01 (m, 1H, H5A), 4.15 (m, 1H, H6A), 4.32 (d, 1H, H8B, J_8A,8B_ = 14.0), 4.84 (d, 1H, H8A, J_8A,8B_ = 14.0), 5.04 (ddd, 1H, H6B, J_6B,5A_ = 3.1, J_6B,5B_ = 7.5, J_6A,6B_ = 15.0), 5.36 (dd, 2H, H10, J_10,11_ = 1.2, J_9(_*_exo_*_),10_ = 4.4), 6.79 (d, 1H, H11, J_10,11_ = 1.2, J_11,12_ = 5.6), 6.83 (d, 1H, H12, J_11,12_ = 5.6), 7.50 (br, 1H, H2, J_1,2_ = J_2,3_ = 7.5), 7.54 (d, 1H, H4, J_3,4_ = 7.5), 7.82 (t, 1H, H3, J_2,3_ = J_3,4_ = 7.5), 7.85 (d, 1H, H1, J_1,2_ = 7.5). Anal. Calcd for C_17_H_18_INO: C, 53.84; H, 4.78; N, 3.69. Found: C, 53.67; H, 4.65; N, 3.62.

### Refinement

The hydrogen atoms were placed in calculated positions with C—H = 0.95–1.00Å and refined in the riding model with fixed isotropic displacement parameters - *U*_iso_(H) = 1.5*U*_eq_(C) for CH_3_-group and *U*_iso_(H) = 1.2*U*_eq_(C) for the other groups.

### Crystal data

**Table:**

C_17_H_18_NO^+^·I^−^	*F*(000) = 1504
*M**_r_* = 379.22	*D*_x_ = 1.648 Mg m^−^^3^
Monoclinic, *C*2/*c*	Melting point: 452 K
Hall symbol: -C 2yc	Mo *K*α radiation, λ = 0.71073 Å
*a* = 15.5047 (6) Å	Cell parameters from 7921 reflections
*b* = 8.0757 (3) Å	θ = 2.7–32.5°
*c* = 25.1874 (12) Å	µ = 2.09 mm^−^^1^
β = 104.204 (1)°	*T* = 100 K
*V* = 3057.3 (2) Å^3^	Prism, orange
*Z* = 8	0.30 × 0.20 × 0.15 mm

### Data collection

**Table:**

Bruker APEXII CCD diffractometer	4450 independent reflections
Radiation source: fine-focus sealed tube	4123 reflections with *I* > 2σ(*I*)
graphite	*R*_int_ = 0.031
φ and ω scans	θ_max_ = 30.0°, θ_min_ = 1.7°
Absorption correction: multi-scan (*SADABS*; Sheldrick, 2003)	*h* = −21→21
*T*_min_ = 0.573, *T*_max_ = 0.745	*k* = −11→11
18881 measured reflections	*l* = −35→35

### Refinement

**Table:**

Refinement on *F*^2^	Primary atom site location: structure-invariant direct methods
Least-squares matrix: full	Secondary atom site location: difference Fourier map
*R*[*F*^2^ > 2σ(*F*^2^)] = 0.025	Hydrogen site location: inferred from neighbouring sites
*wR*(*F*^2^) = 0.054	H-atom parameters constrained
*S* = 1.00	*w* = 1/[σ^2^(*F*_o_^2^) + (0.0195*P*)^2^ + 6.6*P*] where *P* = (*F*_o_^2^ + 2*F*_c_^2^)/3
4450 reflections	(Δ/σ)_max_ = 0.002
182 parameters	Δρ_max_ = 0.48 e Å^−^^3^
0 restraints	Δρ_min_ = −0.94 e Å^−^^3^

### Special details

**Table:**

Geometry. All s.u.'s (except the s.u. in the dihedral angle between two l.s. planes) are estimated using the full covariance matrix. The cell s.u.'s are taken into account individually in the estimation of s.u.'s in distances, angles and torsion angles; correlations between s.u.'s in cell parameters are only used when they are defined by crystal symmetry. An approximate (isotropic) treatment of cell s.u.'s is used for estimating s.u.'s involving l.s. planes.
Refinement. Refinement of *F*^2^ against ALL reflections. The weighted *R*-factor w*R* and goodness of fit *S* are based on *F*^2^, conventional *R*-factors *R* are based on *F*, with *F* set to zero for negative *F*^2^. The threshold expression of *F*^2^ > σ(*F*^2^) is used only for calculating *R*-factors(gt) etc. and is not relevant to the choice of reflections for refinement. *R*-factors based on *F*^2^ are statistically about twice as large as those based on *F*, and *R*-factors based on ALL data will be even larger.

### Fractional atomic coordinates and isotropic or equivalent isotropic displacement parameters (Å^2^)

**Table:**

	*x*	*y*	*z*	*U*_iso_*/*U*_eq_	
I1	0.325921 (8)	0.080703 (16)	0.405918 (5)	0.02178 (4)	
O1	0.45929 (8)	0.66340 (17)	0.33020 (5)	0.0191 (3)	
C1	0.61820 (12)	0.8559 (2)	0.27922 (7)	0.0184 (3)	
H1	0.5871	0.7567	0.2663	0.022*	
C1A	0.63271 (12)	0.9011 (2)	0.33406 (7)	0.0165 (3)	
C2	0.64969 (13)	0.9574 (2)	0.24349 (8)	0.0206 (4)	
H2	0.6392	0.9292	0.2058	0.025*	
C3	0.69669 (13)	1.1005 (2)	0.26325 (8)	0.0226 (4)	
H3	0.7178	1.1700	0.2387	0.027*	
C4	0.71330 (13)	1.1433 (2)	0.31838 (8)	0.0206 (4)	
H4	0.7472	1.2395	0.3314	0.025*	
C4A	0.68027 (12)	1.0454 (2)	0.35434 (8)	0.0183 (3)	
C5	0.69532 (13)	1.0806 (2)	0.41463 (8)	0.0214 (4)	
H5A	0.7106	1.1989	0.4217	0.026*	
H5B	0.7458	1.0134	0.4353	0.026*	
C6	0.61228 (13)	1.0400 (2)	0.43404 (8)	0.0205 (4)	
H6A	0.6255	1.0475	0.4745	0.025*	
H6B	0.5646	1.1204	0.4184	0.025*	
N7	0.58279 (10)	0.8714 (2)	0.41628 (6)	0.0174 (3)	
C8	0.52789 (12)	0.7704 (2)	0.44453 (7)	0.0192 (3)	
H8A	0.4659	0.8114	0.4365	0.023*	
H8B	0.5530	0.7705	0.4847	0.023*	
C8A	0.53246 (12)	0.5980 (2)	0.42045 (7)	0.0175 (3)	
C9	0.44431 (13)	0.4996 (3)	0.40199 (8)	0.0228 (4)	
H9A	0.3945	0.5575	0.4123	0.027*	
H9B	0.4501	0.3866	0.4177	0.027*	
C10	0.43173 (13)	0.4959 (3)	0.33880 (8)	0.0223 (4)	
H10	0.3704	0.4661	0.3174	0.027*	
C11	0.50568 (14)	0.3957 (2)	0.32470 (8)	0.0221 (4)	
H11	0.5008	0.2876	0.3094	0.027*	
C12	0.57905 (12)	0.4890 (2)	0.33795 (7)	0.0187 (3)	
H12	0.6365	0.4647	0.3329	0.022*	
C12A	0.54908 (11)	0.6422 (2)	0.36281 (7)	0.0158 (3)	
C12B	0.59353 (12)	0.8077 (2)	0.37104 (7)	0.0162 (3)	
C13	0.60762 (13)	0.4998 (3)	0.45782 (8)	0.0225 (4)	
H13A	0.5933	0.4803	0.4931	0.034*	
H13B	0.6632	0.5628	0.4637	0.034*	
H13C	0.6146	0.3934	0.4406	0.034*	

### Atomic displacement parameters (Å^2^)

**Table:**

	*U*^11^	*U*^22^	*U*^33^	*U*^12^	*U*^13^	*U*^23^
I1	0.02024 (6)	0.02454 (7)	0.01967 (6)	−0.00275 (5)	0.00320 (4)	−0.00284 (5)
O1	0.0163 (6)	0.0212 (6)	0.0176 (6)	0.0003 (5)	0.0001 (5)	0.0018 (5)
C1	0.0191 (8)	0.0182 (8)	0.0174 (8)	−0.0002 (7)	0.0039 (6)	−0.0004 (7)
C1A	0.0178 (8)	0.0158 (8)	0.0161 (8)	0.0015 (6)	0.0044 (6)	0.0012 (6)
C2	0.0208 (8)	0.0240 (9)	0.0180 (8)	0.0010 (7)	0.0067 (7)	0.0004 (7)
C3	0.0219 (9)	0.0225 (9)	0.0249 (9)	0.0008 (7)	0.0087 (7)	0.0048 (7)
C4	0.0193 (8)	0.0174 (8)	0.0256 (9)	−0.0007 (7)	0.0064 (7)	−0.0006 (7)
C4A	0.0163 (8)	0.0177 (8)	0.0199 (8)	0.0007 (6)	0.0028 (6)	−0.0009 (6)
C5	0.0230 (9)	0.0215 (9)	0.0193 (8)	−0.0031 (7)	0.0043 (7)	−0.0033 (7)
C6	0.0268 (9)	0.0176 (8)	0.0183 (8)	−0.0021 (7)	0.0079 (7)	−0.0035 (7)
N7	0.0200 (7)	0.0164 (7)	0.0162 (7)	−0.0001 (6)	0.0051 (6)	0.0003 (6)
C8	0.0213 (8)	0.0213 (9)	0.0161 (8)	−0.0003 (7)	0.0066 (6)	0.0006 (7)
C8A	0.0178 (8)	0.0191 (8)	0.0157 (8)	−0.0016 (6)	0.0045 (6)	0.0011 (6)
C9	0.0215 (9)	0.0265 (10)	0.0209 (9)	−0.0070 (7)	0.0058 (7)	0.0003 (8)
C10	0.0199 (8)	0.0245 (10)	0.0217 (9)	−0.0063 (7)	0.0032 (7)	−0.0005 (7)
C11	0.0273 (9)	0.0190 (9)	0.0191 (8)	−0.0031 (7)	0.0038 (7)	−0.0008 (7)
C12	0.0206 (8)	0.0184 (8)	0.0172 (8)	0.0014 (7)	0.0046 (7)	−0.0002 (7)
C12A	0.0152 (7)	0.0171 (8)	0.0151 (7)	−0.0011 (6)	0.0034 (6)	0.0010 (6)
C12B	0.0167 (8)	0.0164 (8)	0.0147 (7)	0.0013 (6)	0.0021 (6)	0.0006 (6)
C13	0.0246 (9)	0.0242 (10)	0.0178 (8)	0.0029 (7)	0.0036 (7)	0.0047 (7)

### Geometric parameters (Å, °)

**Table:**

O1—C12A	1.443 (2)	N7—C8	1.481 (2)
O1—C10	1.451 (2)	C8—C8A	1.527 (3)
C1—C2	1.391 (3)	C8—H8A	0.9900
C1—C1A	1.393 (2)	C8—H8B	0.9900
C1—H1	0.9500	C8A—C13	1.528 (3)
C1A—C4A	1.406 (3)	C8A—C9	1.550 (3)
C1A—C12B	1.443 (2)	C8A—C12A	1.576 (2)
C2—C3	1.391 (3)	C9—C10	1.555 (3)
C2—H2	0.9500	C9—H9A	0.9900
C3—C4	1.392 (3)	C9—H9B	0.9900
C3—H3	0.9500	C10—C11	1.515 (3)
C4—C4A	1.392 (3)	C10—H10	1.0000
C4—H4	0.9500	C11—C12	1.337 (3)
C4A—C5	1.506 (3)	C11—H11	0.9500
C5—C6	1.521 (3)	C12—C12A	1.510 (3)
C5—H5A	0.9900	C12—H12	0.9500
C5—H5B	0.9900	C12A—C12B	1.495 (3)
C6—N7	1.470 (2)	C13—H13A	0.9800
C6—H6A	0.9900	C13—H13B	0.9800
C6—H6B	0.9900	C13—H13C	0.9800
N7—C12B	1.298 (2)		
			
C12A—O1—C10	94.73 (13)	C8—C8A—C13	109.33 (15)
C2—C1—C1A	119.36 (18)	C8—C8A—C9	117.58 (16)
C2—C1—H1	120.3	C13—C8A—C9	113.69 (16)
C1A—C1—H1	120.3	C8—C8A—C12A	101.17 (14)
C1—C1A—C4A	121.36 (17)	C13—C8A—C12A	114.35 (15)
C1—C1A—C12B	120.78 (17)	C9—C8A—C12A	99.85 (14)
C4A—C1A—C12B	117.70 (16)	C8A—C9—C10	101.35 (14)
C1—C2—C3	119.60 (18)	C8A—C9—H9A	111.5
C1—C2—H2	120.2	C10—C9—H9A	111.5
C3—C2—H2	120.2	C8A—C9—H9B	111.5
C2—C3—C4	121.04 (18)	C10—C9—H9B	111.5
C2—C3—H3	119.5	H9A—C9—H9B	109.3
C4—C3—H3	119.5	O1—C10—C11	101.25 (15)
C4A—C4—C3	120.02 (18)	O1—C10—C9	99.63 (15)
C4A—C4—H4	120.0	C11—C10—C9	109.76 (16)
C3—C4—H4	120.0	O1—C10—H10	114.8
C4—C4A—C1A	118.58 (18)	C11—C10—H10	114.8
C4—C4A—C5	123.94 (17)	C9—C10—H10	114.8
C1A—C4A—C5	117.44 (17)	C12—C11—C10	106.70 (17)
C4A—C5—C6	110.45 (16)	C12—C11—H11	126.7
C4A—C5—H5A	109.6	C10—C11—H11	126.7
C6—C5—H5A	109.6	C11—C12—C12A	103.62 (16)
C4A—C5—H5B	109.6	C11—C12—H12	128.2
C6—C5—H5B	109.6	C12A—C12—H12	128.2
H5A—C5—H5B	108.1	O1—C12A—C12B	108.67 (14)
N7—C6—C5	109.06 (15)	O1—C12A—C12	102.32 (14)
N7—C6—H6A	109.9	C12B—C12A—C12	127.70 (16)
C5—C6—H6A	109.9	O1—C12A—C8A	101.44 (13)
N7—C6—H6B	109.9	C12B—C12A—C8A	104.40 (14)
C5—C6—H6B	109.9	C12—C12A—C8A	109.46 (15)
H6A—C6—H6B	108.3	N7—C12B—C1A	121.63 (17)
C12B—N7—C6	122.46 (16)	N7—C12B—C12A	108.79 (15)
C12B—N7—C8	114.58 (16)	C1A—C12B—C12A	129.10 (16)
C6—N7—C8	122.45 (15)	C8A—C13—H13A	109.5
N7—C8—C8A	102.91 (14)	C8A—C13—H13B	109.5
N7—C8—H8A	111.2	H13A—C13—H13B	109.5
C8A—C8—H8A	111.2	C8A—C13—H13C	109.5
N7—C8—H8B	111.2	H13A—C13—H13C	109.5
C8A—C8—H8B	111.2	H13B—C13—H13C	109.5
H8A—C8—H8B	109.1		
			
C2—C1—C1A—C4A	−1.4 (3)	C10—C11—C12—C12A	2.8 (2)
C2—C1—C1A—C12B	173.85 (17)	C10—O1—C12A—C12B	−169.84 (14)
C1A—C1—C2—C3	1.3 (3)	C10—O1—C12A—C12	52.90 (15)
C1—C2—C3—C4	0.4 (3)	C10—O1—C12A—C8A	−60.19 (15)
C2—C3—C4—C4A	−2.1 (3)	C11—C12—C12A—O1	−35.95 (18)
C3—C4—C4A—C1A	2.0 (3)	C11—C12—C12A—C12B	−161.61 (17)
C3—C4—C4A—C5	179.45 (18)	C11—C12—C12A—C8A	71.05 (18)
C1—C1A—C4A—C4	−0.2 (3)	C8—C8A—C12A—O1	−86.84 (15)
C12B—C1A—C4A—C4	−175.63 (16)	C13—C8A—C12A—O1	155.78 (15)
C1—C1A—C4A—C5	−177.87 (17)	C9—C8A—C12A—O1	34.03 (17)
C12B—C1A—C4A—C5	6.7 (2)	C8—C8A—C12A—C12B	26.06 (17)
C4—C4A—C5—C6	142.02 (19)	C13—C8A—C12A—C12B	−91.31 (18)
C1A—C4A—C5—C6	−40.5 (2)	C9—C8A—C12A—C12B	146.94 (15)
C4A—C5—C6—N7	51.2 (2)	C8—C8A—C12A—C12	165.56 (14)
C5—C6—N7—C12B	−32.4 (2)	C13—C8A—C12A—C12	48.2 (2)
C5—C6—N7—C8	156.17 (16)	C9—C8A—C12A—C12	−73.57 (17)
C12B—N7—C8—C8A	20.2 (2)	C6—N7—C12B—C1A	−2.2 (3)
C6—N7—C8—C8A	−167.72 (16)	C8—N7—C12B—C1A	169.88 (16)
N7—C8—C8A—C13	94.15 (16)	C6—N7—C12B—C12A	−174.87 (16)
N7—C8—C8A—C9	−134.27 (16)	C8—N7—C12B—C12A	−2.8 (2)
N7—C8—C8A—C12A	−26.83 (17)	C1—C1A—C12B—N7	−158.84 (18)
C8—C8A—C9—C10	112.08 (18)	C4A—C1A—C12B—N7	16.6 (3)
C13—C8A—C9—C10	−118.35 (17)	C1—C1A—C12B—C12A	12.3 (3)
C12A—C8A—C9—C10	3.87 (18)	C4A—C1A—C12B—C12A	−172.30 (17)
C12A—O1—C10—C11	−50.15 (15)	O1—C12A—C12B—N7	92.29 (17)
C12A—O1—C10—C9	62.39 (15)	C12—C12A—C12B—N7	−144.62 (18)
C8A—C9—C10—O1	−40.39 (17)	C8A—C12A—C12B—N7	−15.34 (19)
C8A—C9—C10—C11	65.35 (19)	O1—C12A—C12B—C1A	−79.7 (2)
O1—C10—C11—C12	30.66 (19)	C12—C12A—C12B—C1A	43.4 (3)
C9—C10—C11—C12	−74.0 (2)	C8A—C12A—C12B—C1A	172.67 (17)

### Hydrogen-bond geometry (Å, °)

**Table:**

*D*—H···*A*	*D*—H	H···*A*	*D*···*A*	*D*—H···*A*
C2—H2···I1^i^	0.95	3.23	3.999 (2)	139
C6—H6A···I1^ii^	0.99	3.10	4.028 (2)	157
C8—H8A···I1^iii^	0.99	3.04	3.942 (2)	153
C8—H8B···I1^ii^	0.99	3.17	4.072 (2)	153
C9—H9B···I1	0.99	3.10	3.862 (2)	135
C12—H12···I1^iv^	0.95	3.20	3.862 (2)	128
C13—H13B···I1^iv^	0.98	3.21	3.970 (2)	136

Symmetry codes: (i) −*x*+1, *y*+1, −*z*+1/2; (ii) −*x*+1, −*y*+1, −*z*+1; (iii) *x*, *y*+1, *z*; (iv) *x*+1/2, *y*+1/2, *z*.
